# Linkage of the Avon Longitudinal Study of Parents and Children (ALSPAC) to Avon & Somerset Police regional police records

**DOI:** 10.12688/wellcomeopenres.18720.1

**Published:** 2023-02-01

**Authors:** Alison Teyhan, Rosie Cornish, Andy Boyd, Richard Thomas, Mark Mumme, Amy Dillon, Iain Brennan, Adrian Brown, Anna Ferrante, John Macleod

**Affiliations:** 1 ALSPAC, Population Health Sciences, Bristol Medical School, University of Bristol, Bristol, BS8 2BN, UK; 2 MRC Integrative Epidemiology Unit, Bristol Medical School, University of Bristol, Bristol, BS8 2BN, UK; 3 UK Longitudinal Linkage Collaboration, Population Health Sciences, Bristol Medical School, University of Bristol, Bristol, BS8 2PS, UK; 4 NIHR Biomedical Research Centre Bristol, University Hospitals Bristol and Weston NHS Foundation Trust, University of Bristol, Bristol, BS8 2BN, UK; 5 School of Criminology, Sociology and Policing, University of Hull, Hull, HU6 7RX, UK; 6 Centre for Data Linkage, Curtin University, Perth, Australia; 7 National Institute for Health Research Applied Research Collaboration West (NIHR ARC West), University Hospitals Bristol and Weston NHS Foundation Trust, Bristol, BS1 2NT, UK; 8 Centre for Academic Primary Care, Population Health Sciences, University of Bristol, Bristol, BS8 2PS, UK

**Keywords:** ALSPAC, birth cohort, police data, linkage, crime

## Abstract

This data note describes a new resource for crime-related research: the Avon Longitudinal Study of Parents and Children (ALSPAC) linked to regional police records. The police data were provided by Avon & Somerset Police (A&SP), whose area of responsibility contains the ALSPAC recruitment area. In total, ALSPAC had permission to link to crime records for 12,662 of the ‘study children’ (now adults, who were born in the early 1990s).  The linkage took place in two stages: Stage 1 involved the ALSPAC Data Linkage Team establishing the linkage using personal identifiers common to both the ALSPAC participant database and A&SP records using deterministic and probabilistic methods. Stage 2 involved A&SP extracting attribute data on the matched individuals, removing personal identifiers and securely sharing the de-identified records with ALSPAC. The police data extraction took place in July 2021, when the participants were in their late 20s/early 30s. This data note contains details on the resulting linked police records available. In brief, electronic police records were available from 2007 onwards. In total, 1757 participants (14%) linked to at least one police record for a charge, offence ‘taken into consideration’, caution, or another out of court disposal. Linked participants had a total of 6413 records relating to 6283 offences. Almost three quarters of the linked participants were male. The most common offence types were violence against the person (22% of records), drug offences (19%), theft (17%) and public order offences (11%). This data note also details important issues that researchers using the local police data should be aware of, including the importance of defining an appropriate denominator, completeness, and biases affecting police records.

## Background

A public health approach to tackling crime means a focus on populations rather than individuals, proactive prevention, and the tackling of upstream risk factors ^1, 2^. To be successful, this approach relies on suitable data to produce a strong evidence base that can inform the design and delivery of effective interventions ^1^. Police records alone cannot be used for this purpose as they do not contain data relating to an individual’s exposure to potential risk factors for perpetrating crime. However, the linkage of police records to longitudinal cohort study data has the potential to create a data resource that could be used to study both the antecedents and consequences of involvement with the criminal justice system. This is because many longitudinal birth cohort studies have detailed measures of the lives of their participants, and often their families, peers, and wider contexts, across the life course.

One such study is the Avon Longitudinal Study of Parents and Children (ALSPAC). It began in the early 1990s and the study children are now adults. ALSPAC has already established linkages to participants’ routinely collected electronic health, education, and geographic records. With regard to criminality records, ALSPAC had planned to link to the Police National Computer (PNC), which is a large centralised administrative database maintained by the Ministry of Justice (MoJ) that was started in 1974 and contains information about police cautions and court convictions in England and Wales ^3^. A pilot linkage based on an anonymised extract of PNC data was achieved in 2013 ^4^; however, this did not progress to a full linkage. A finding of the pilot study was that the majority of offences committed by the ALSPAC participants (86%) took place in the policing area local to ALSPAC (Avon and Somerset). It was therefore decided that pursuing linkage to local police records would be a more targeted yet equally valid approach. 

The linkage of ALSPAC to Avon and Somerset Police (A&SP) records is the focus of this data note. The aims are: 1. to detail the linkage process, 2. to describe the police data available, 3. to highlight important considerations and limitations of the police data.

## Materials and methods

### Data sources

***Avon Longitudinal Study of Parents and Children (ALSPAC).*** ALSPAC began with the recruitment of pregnant women who had an expected due date between April 1991 and December 1992 and who lived in a defined area in and around the city of Bristol, UK. The precise geographical catchment is described elsewhere ^5^ and broadly matches what are the present-day counties of Bristol, North Somerset and South Gloucestershire. There were 13,988 study children alive at one year of age. An additional 718 children, who met the original study eligibility criteria, but whose mothers had not joined the study during pregnancy, were recruited by age 18 years. Full details on ALSPAC are given in the cohort profiles ^5, 6^ and the study website contains details of all the data available through a fully searchable data dictionary and variable search tool. 

Ethical approval for the study was obtained from the ALSPAC Ethics and Law Committee (ALEC) and the Local Research Ethics Committees. Study involvement of the index participants (the children born in 1991–92) was based on parental approval until the children reached adulthood. Informed consent for the use of data collected via questionnaires and clinics was obtained from participants following the recommendations of ALEC at the time. When participants reached age 18 years they were sent ‘fair processing’ materials that invited them to continue to take part in ALSPAC and informed them about ALSPAC’s intention to link to their routine health and administrative records, including any criminality records, and gave a clear means to opt out. Where practicable ( *e.g.* when attending a study assessment visit), participants were also able to explicitly consent. The original materials referred to linkage to PNC records. Therefore, before the linkage to regional police records held by A&SP, an update to the fair processing materials was provided to participants, using both online and postal materials, with a clear means to opt out. At the time of the linkage (July 2021) we had permission to link to the criminality records of 12,662 participants (comprising 5,055 who had explicitly opted-in to criminality linkage and 7,607 who had received the opt-out linkage form and did not respond). Participants who opted out of linkage to criminality records (4% to date), or who did not receive fair processing materials, were not included in the linkage to A&SP records. 

***Avon and Somerset Police.*** A&SP are responsible for law enforcement in the four counties that replaced the now abolished county of Avon (Bristol, Bath and North-East Somerset, North Somerset, and South Gloucestershire), plus the county of Somerset. The A&SP area (population 1.73 million; area 4784km ^2 ^) therefore includes the full ALSPAC recruitment area and some neighbouring areas.

Offences committed in the Avon and Somerset area, and which come to the attention of the police, are recorded by A&SP in their database. Offences in other areas of the country, or abroad, are not recorded in their database. The numbers of crimes recorded annually by A&SP, and every other police force in England and Wales, are available online ^7^. 

A&SP have used the NicheRMS365 cloud platform as their record management system since September 2015. Older electronic records (from the Guardian system that predated Niche) have been migrated to the Niche platform. Pre-2007, a large proportion of records were held on a system called CMU2 and these only held an electronic reference for the offence, with the majority of the information being held in a paper format. Some of these paper records are still held in the force archive and are researched as part of the police review process (detailed in next paragraph) and, if relevant for retention or if they add value to the record, the force’s Retention, Review and Disposal (RRD) Team can scan and upload them to Niche. 

The Management of Police Information (MoPI) Code of Practice gives guidance in relation to the review, retention and disposal of policing information and records. Police records must be regularly reviewed to ensure that they remain necessary for a policing purpose, are accurate, adequate and up-to-date, and are kept for no longer than is necessary. Details of the review process are available online. In brief, all offences are categorised into one of three MoPI groups. Group 1 offences are the most serious, and Group 2 covers sexual, violent and serious offences not included in Group 1. The MoPI guidance is that Group 1 and 2 records are reviewed after a 10 year clear period ( *i.e.* a 10 year period in which the offender has not come to the police’s notice again). Group 1 and 2 records can only be deleted from the police database if that is deemed appropriate after a manual review process conducted by the RRD Team. If, for example, the subject is deemed to pose a high risk of harm, records would be retained and reviewed after a further 10 year clear period. Group 3 covers all other offences. These records are reviewed after an initial six year clear period and then, if retained, reviewed again every subsequent five year clear period. These records are currently manually reviewed and deleted as appropriate, but this could be automated for suitable Group 3 records in the future. All pre-2007 A&SP records relating to MoPI Group 3 offences have been manually reviewed and disposed of. Note that where a person is linked to multiple offences, the most serious offence determines the review category for all offences.

## Linkage methodology

Data Processing Agreements for the transfer of A&SP data to ALSPAC were finalised in June 2020, and the police data were extracted in July 2021. The linkage took place in two stages, detailed below. All data processing was conducted by the three data managers in the ALSPAC Data Linkage Team, all of whom were individually security cleared by A&SP prior to the commencement of this project. All data processing took place within the ALSPAC Data Safe Haven, which is accredited to the ISO27001 information security standard.

### Stage 1: Using personal identifiers to establish matches

As there is no strong, persistent identifier common to both ALSPAC and the A&SP dataset, a number of personal data items available in both datasets were used to determine which individuals in ALSPAC had an A&SP record. A&SP sent ALSPAC the forename, surname, date of birth (DoB), sex and full current and historical address(es) of all individuals held in their database who were born between 1 ^st^ January 1991 and 31 ^st^ January 1993 (the date range in which the vast majority of ALSPAC study children were born). No information about these individuals was sent other than these identifiers and a unique record ID ( *‘offender_id’*). Comparable identifiers were extracted from the ALSPAC participant database for the 12,662 participants for whom ALSPAC had permission to link to criminal record data.

A combination of deterministic and probabilistic record linkage methods were used to maximise linkage coverage and minimise false matches. Firstly, a deterministic match was completed using forename, surname, and DoB ( *i.e.* these identifiers needed to be identical for there to be a ‘match’). This yielded 1876 matches to unique A&SP ‘ *offender_ids’*. Postcode was then used to create a match strength variable ( Table 1). Postcode was not included as a mandatory matching variable as it was considered likely that ALSPAC participants with a criminality record would be less likely to be actively engaged in the study (as both criminal involvement ^8, 9^ and ALSPAC participation ^4, 10^ are associated with social position), meaning the address information ALSPAC had for them at the time of linkage may have been out of date.

**Table 1.** Deterministic match criteria and number of matches.

Matching Criteria	Match strength	Number of matches
Forename, surname, DoB, full postcode ^1^	1	956
Forename, surname, DoB, first part of postcode ^1^	2	403
Forename, surname, DoB	3	517
		**1876 TOTAL**

^1^A postcode in the UK consists of two alphanumeric codes – the first part identifies the post town, and the second part relates to a few addresses within that post town (usually a group of around 15).

**Figure 1.**
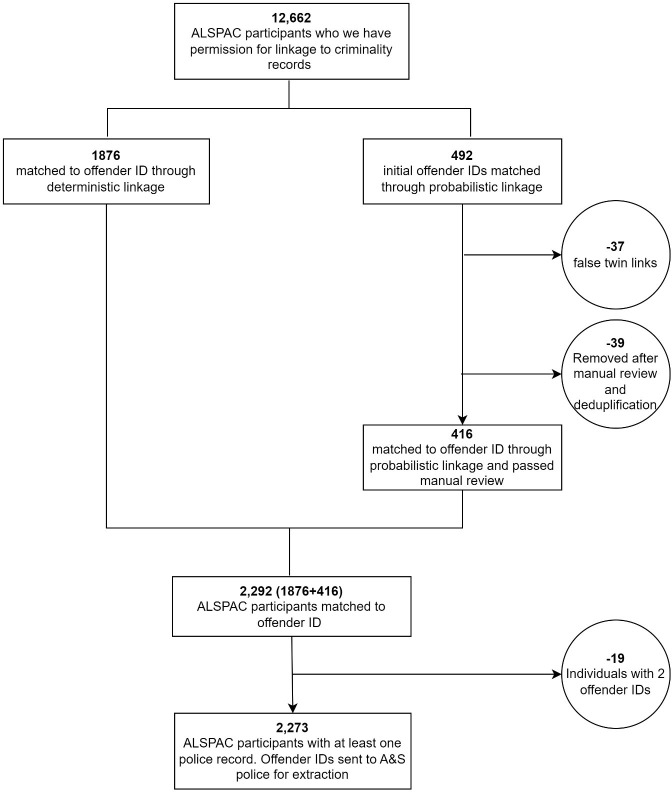
Flow chart of linkage of ALSPAC participants to Offender IDs.

Probabilistic linkage was then used, which uses conditional probabilities to compute likelihood estimates for each field. Record comparisons involve comparisons for each field and the sum of the weights determined by each field comparison (using the likelihood estimate) provide an overall score. Record comparison scores over a defined threshold are designated a match. This probabilistic approach, along with the use of similarity comparisons, allows variations such as full name and short form names, errors in postcode details and typographical errors. The probabilistic matching procedure was conducted using the default settings in the LinXmart record linkage software (Version 1.8.3) developed by the Centre for Data Linkage at Curtin University, Australia ^11^. LinXmart is able to perform linkage across event-level datasets, based on user-defined matching of demographic information using a probabilistic approach. (The authors used LinXmart free of charge based on our collaboration with Curtin University, but it is also more widely available as a commercial product. There are various open-access alternatives, including Splink). This method yielded 2292 matches to unique A&SP ‘ *offender_ids’*. This included all 1876 linked using the deterministic linkage process plus an additional 416 matched through probabilistic linkage and who passed two manual review stages (detailed in next paragraph). Therefore, in total 2273 ALSPAC individuals who had at least one record in the A&SP dataset were identified ( Figure 1). 

The first manual review of the links achieved with probabilistic methods was a ‘twin check’; as twins share a number of common identifiers, they are at high risk of creating a false link. A list of twins (from ALSPAC administrative data) was used to create a flag to highlight individuals to be checked. These were required to match the A&SP identifiers on both gender and the first two characters of forename. This resulted in 37 *offender_ids* being removed. The second manual review used the LinXmart generated metric that indicates matching confidence between records. Those in the bottom 10% (lowest confidence) were selected for manual review and the following rules were derived that required these records to have:

- A match on at least forename AND full date of birth
- OR a surname AND full date of birth match
- OR full postcode match

This resulted in an additional 34 individuals being removed from the linkage file. These individuals were heavily concentrated towards the lowest confidence scores. A few 'true' links also had some of the lowest confidence scores, indicating that the manual review was a worthwhile exercise (as opposed to setting a fixed/hard confidence threshold).

Duplicate checks were then performed. These identified three *offender_ids* matching to the same individual. These were removed, as none of these records had DoB or postcode matches, and the names alone were not deemed to have sufficient distinguishing power to confirm a match. Further, a duplicate check was run on the *offender_id* field and four duplicates were identified. Only the *offender_id* with a strong match (based on postcode) was retained. In total, 39 offender IDs were removed during the second manual review and de-duplication step. 

It was also found that 19 ALSPAC individuals each linked to two *offender_ids* ( *i.e.* the police had marked an individual as two different people in their database, but they were the same person according to the ALSPAC database). In these cases, records belonging to both *offender_ids* were retained and linked to the same ALSPAC individual.

At the end of the linkage process, all personal identifiers provided by A&SP were securely destroyed in line with ALSPAC’s ISO27001 certified processes. This left an ID match variable (ALSPAC ID to A&SP *offender_ids*) and linkage quality variables.

### Stage 2: Extracting attribute data

A&SP extracted 11,681 de-identified police event records related to the 2,273 individuals matched in Stage 1 and securely transferred these records to ALSPAC. In this event-based dataset, each row is a record that corresponds to a crime occurrence for an individual. A&SP records include the disposal outcome(s) for each crime. Of the disposal outcome types available in the police dataset, ALSPAC has an ethico-legal basis (set through the participant fair processing) to link to records with the following types: charges, crimes ‘taken into consideration’ (TICs), cautions, and other out-of-court disposals (penalty notices, drug warnings, and community resolutions). The threshold of evidence needed for an individual to be charged is high ^12^, and the majority will go on to face trial in court. Conviction rates are high for many offences, but do vary by offence type ^13^. TICs are crimes taken into consideration at the time of sentencing for another crime. The individual may volunteer these offences, or they may be asked by the police if they accept them. In either case, the individual must formally admit guilt to the additional crime(s) while under caution. To be issued with an out-of-court disposal, an individual must admit they are guilty of the offence and be eligible in terms of previous recorded offending (these disposals are designed to be used in situations of low-level offending). Notably, and in contrast to the PNC, A&SP do not routinely record conviction data. 

The process to identify which A&SP records had an eligible disposal type was complicated by the fact that the police record many details of crimes at an offence level rather than at a person (offender) level. A&SP provided a variable which states how many offenders were involved in each crime; this enabled identification of ‘group crimes’, *i.e.* a single recorded crime that was alleged to have been perpetrated by multiple offenders. The following outcome variables were then used to determine the disposal type:

- For crimes involving one offender: the main outcome variable, *Currentclassificationhooutcom,* was used. This is a 22 category variable that gives the Home Office outcome code for each offence ^14^. This is an offence-level variable, but for offences that involve only one offender, it is in effect an individual-level variable. Records were linked to ALSPAC if *currentclassificationhooutcom* was OC1 (charged), OC2-3 (cautioned), OC4 (taken into consideration), OC6 (penalty notice for disorder), OC7 (cannabis warning), or OC8 (community resolution).
- For crimes involving multiple offenders (‘group crimes’): for offences involving more than one offender, *Currentclassificationhooutcom* cannot be used as it is an offence-level variable, meaning everyone with a record for that offence is assigned the same outcome, the most serious outcome of the group. Instead, a secondary outcome variable *offenderclassificationconcat* was used. This is an individual-level, concatenated variable which lists several terms (up to six) for each individual ( *e.g.* ‘suspect; arrested; charged’). Note that prior to September 2015 (when police recording software changed to Niche), the term ‘prosecuted’ was used in the concatenated variable to cover TICs and all out-of-court disposals (cautions, penalty notices, drug warnings, and community resolutions). Records were linked to ALSPAC if *offenderclassificationconcat* contained at least one of the following terms (which relate to OC 1-4 and 6-8): charged, TIC, cautioned, adult conditional caution, postal requisition, reported for summons, cannabis warning, penalty notice for disorder, community resolution, prosecuted.

**Figure 2.**
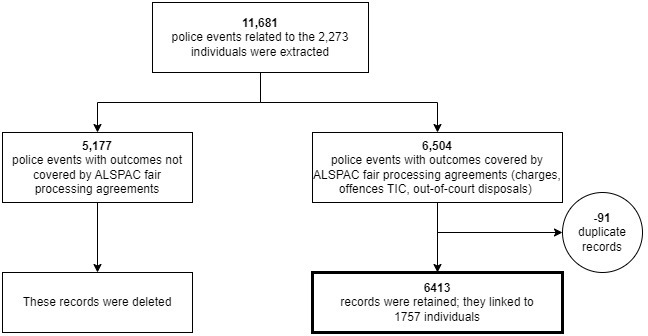
Flow chart of linkage of police events records to ALSPAC participants.

**Figure 3.**
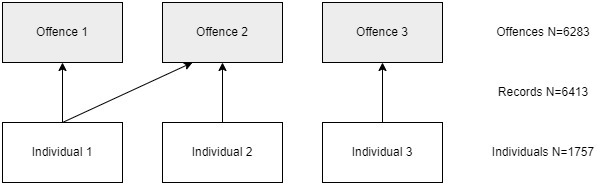
Relationship between individuals, crime records and offences.

All other records were deleted (these include records where the individual had been eliminated from enquiries, or where there was insufficient evidence to proceed). This resulted in a final sample of 6413 police records ( Figure 2). The 6413 records relate to 1757 individuals and 6283 separate offences (an example of the data structure is shown in Figure 3).

## The A&S Police dataset

### Data provided by A&SP

The A&SP data set contains 19 variables. These comprise administrative variables, date variables that specify when an offence took place and when it was reported to police, type and severity of offence variables, disposal type variables, flag variables, and variables related to Magistrates’ Court appearances. Table 2 lists the variable names along with a brief description, a summary of the missing data, and a note as to whether they are available to researchers.

**Table 2.** Variables in the dataset provided by A&S police.

Variable type	Variable name	Description	% missing (100%=6413)	Available for researchers?
Administrative	occurrence_id	ID of the crime	0%	Yes ^1^
	offendercount	How many offenders were involved in the crime	0%	Yes
Date	occurrencecreateddate	System generated, triggered by a 111/999 call about an occurrence that the officer later declares a crime, or similar.	0%	No (however, age available) ^2^
	occurrencereporteddate	Automatically entered when the crime occurrence is created (generated from STORM ^1^ and pushed to Niche ^2^).	0%	No (however, age available) ^2^
	occurrencefromdate	Date of the offence, person reported via 111/999 or any other way	0.1%	No (however, age available) ^2^
Type/severity of offence	currentoffencegroup	12 category variable giving type of offence	0%	Yes
	currentoffencehocode	Offence Home Office code	0%	No ^3^
	currentoffencedescription	Offence description	0%	No ^3^
	scorexmultiplier	Crime severity score	0%	Yes
Disposal type	currentclassificationhooutcom	Offence-level. Home office outcome code and description	0%	No
	offenderclassificationconcat	Individual-level. String variable with up to 6 terms. This has been split into 6 separate variables.	0%	No
Flag	domesticabuseindicator	Crime involved domestic abuse (no/yes)	0%	Yes
	knifecrimeindicator	Crime involved a knife (no/yes)	0%	Yes
	drugsflagged	Crime involved drugs (no/yes)	0%	Yes
	alcohol	Crime involved alcohol	98.2%	No ^4^
	currentsubstanceusedbyoffend	Offender affected by: alcohol; alcohol and drugs; drugs; not affected; not known. This flag started being used in the mid-2000s but has since fallen into disuse. Not mandatory field.	95.2% (99.0% if not known category is treated as missing)	No ^4^
Magistate’s Court	casefileid	ID of Magistrates’ court case	87.9%	Yes ^1^
	casefilecreateddateandtime	Date of court case	88.2% (3.0% of those with a casefileid)	No (however, age available) ^2^
	verdict	Verdict of Magistrates’ court case (Not guilty; guilty)	92.5% (37.9% of those with a casefileid)	No ^4^

^1^A pseudonymised version of these variables is available. ^2^Age in months has been derived for each of the date variables. ^3^The Home Office code, and corresponding description, variables are not available to researchers due to a large number of codes having small numbers of records. However, researchers can specify an aggregated variable - this will be available provided numbers in each grouping are adequate. ^4^These variables will not be released due to a high proportion of missing data.

With regards the date variables, for over half (53%) of the records *occurrencefromdate* is equal to *occurrencereporteddate* ( *i.e.* the crime was reported on the same day that it occurred). For the records with non-matching dates, the difference ranges from one day to several years: 45% of these records have a difference of only one day (meaning the crime was reported the day after it was thought to have occurred), 73% have a difference of <10 days, and 7% have a difference of over a year. In general, reasons for short time discrepancies between when a crime occurs and the date it is reported to police can include a person not being aware of the exact date of the offence ( *e.g.* house was burgled when on holiday). Reasons for longer discrepancies can include historical sexual assaults, or a catalogue of domestic abuse incidents being reported in one report. 

There are four ‘flag’ variables which specify if a crime involved domestic abuse, knife crime, drugs, or alcohol. There is an additional variable which specifies whether an offender was using drugs and/or alcohol ( *currentsubstanceusedbyoffend*) but this has very high levels of missing data as it is no longer used by the police in their reporting.

The nature of the offence is given by: *currentoffencehocode* (the Home Office code for the offence ^15^); *currentoffencedescription* (a detailed categorical variable which describes these codes); and *currentoffencegroup* (a categorical variable which assigns each of the offences to one of 12 broader offence groups). For example, *currentoffencedescription* describes a code as ‘possession of cannabis’ and *currentoffencegroup* assigns that offence to the ‘drug offences’ category. 

The variable *scorexmultiplier* indicates each offence’s severity. These scores are used by A&SP to monitor the harm arising from crimes as opposed to just measuring crime volume, enabling them to identify the most high-risk offenders and most vulnerable victims. These scores are not used by the courts. The *scorexmultiplier* value is derived from the ‘harm score’ for the offence, increased by a ‘multiplier’ if relevant. Each Home Office offence code has a corresponding harm score, ranging from 0.01 to 100. Offences with a harm score <3 include intent to supply class A drugs (harm score of 0.8), wounding with intent to do serious bodily harm (1.45), and rape (2.9). No offences have a harm score between 3 and 8. Crimes with scores ≥8 include conspiring to traffic a person into the UK for exploitation (8), causing or inciting child pornography (10), manslaughter (30), use of noxious substance in terrorism offence (50), and murder (100). These harm scores are increased by a multiplier if the following factors are present: +30% for domestic abuse related, +50% for hate related, +5% for drug related, +10% if there is a firearm tag, and +30% if there is a safeguarding children tag. If more than one of these factors is present, the multipliers are cumulative and applied in the order listed. Note that ALSPAC has not been provided with variables that specify which multipliers were used in the calculation of each crime’s *scorexmultiplier* value. 

The final three variables relate to Magistrates’ Court appearances (from November 2015 only): *casefileid* is the ID for that court appearance, *casefilecreateddateandtime* gives the date of the court case, and *verdict* states whether the defendant was found guilty or not guilty (this variable has high levels of missing data: this information is generated by the local Crown Prosecution Service, not the police, and the data flow between them can be poor).

### Changes made to A&SP data by ALSPAC

The ALSPAC Data Linkage managers made changes to some of the police data to prevent disclosure of ALSPAC participants’ identities during research use. The changes are:

•   The *occurrence_id* and *casefileid* variables have been pseudonymised but retain equivalent functionality.

•   The date variables will not be released. Instead, the age of the participant on each of these dates has been calculated (in months) using their date of birth. Month and year of offence will be available. 

•   The original outcomes variables ( *currentclassificationhooutcom* and *offenderclassificationconcat*) will not be released. A binary variable has been derived (participant has a police record, yes or no). This binary variable ensures all ALSPAC participants with a record are treated equally (as details on type of disposal are not available for individuals involved in group crimes prior to September 2015, as described above). 

•   The variables that describe the nature of the offence in detail ( *currentoffencehocode* and *currentoffencedescription)* will not be released to researchers in their original format as they have many categories with small cell counts. The offence group variable ( *currentoffencegroup*) will be available. If required, researchers can discuss with the ALSPAC Data Linkage Team options for grouping the Home Office codes in a different way to that available in the *currentoffencegroup* variable.

•   The *scorexmultiplier* and *offendercount* variables will be aggregated at the upper end due to small numbers of records with high scores.

•   Variables with high levels of missing data will not be released.

## Brief summary of the police data available

This section gives a brief overview of the police data, including the time period that the records cover, and the numbers of offences by type of offence and sex. Researchers requiring more detailed information in order to determine if these data are suitable for their research purposes should contact the ALSPAC Data Linkage Team.

**Figure 4.**
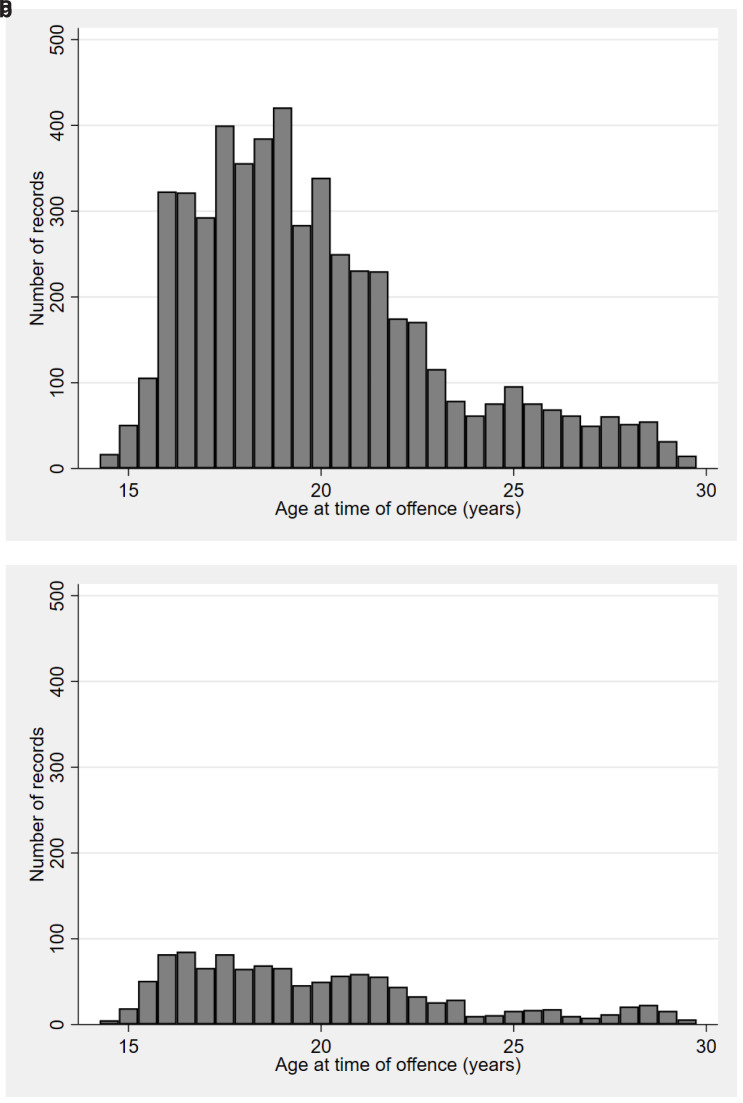
Distribution of age at offence by sex. (Figure 4 **a** is Males, 4 **b** is Females). *Footnote for Figure 4:* Due to small numbers of offences at the youngest and oldest ages, any offences below the age of 14.5 are included in the 14.5 group, and any offences over the age of 29.5 are included in the 29.5 group.

Of those in the ALSPAC sample with an A&SP record, 73% are male. Over three quarters of records are for an offence involving only one person. The years 2009–2010 saw the largest number of offences (when the participants would have been in their late teens). This peak was driven largely by males’ offending ( Figure 4): females have considerably fewer records, and the distribution of their records by year of offence is flatter. Most of the individuals with a record have a small number of records: 47% have one, and 18% have two (range 1 to >150, median 2). For those with more than one record, the time difference between first and last offence ranges from 0 (i.e. all offences took place on same day) to several years (median 3.9 years). Note that the police data are left and right censored (few records available pre-2007 as they were not in electronic format, and no records for offences that were reported after the linkage to ALSPAC occurred in July 2021). In terms of crime severity, the *scorexmultiplier* variable has a range of 0.01 to over 100, with most records having a relatively low score (74% of records have a severity score of ≤0.2). 

The 6413 A&SP records linked to ALSPAC cover a wide range of offence groups. The most common groups were violence against the person (22% of records), drug offences (19%), theft (17%) and public order offences (11%) ( Table 3). All offences were more common in males than females. There are similarities and differences in the distribution of offences by sex. For example, violent crime accounts for a similar percentage of the crimes committed by males and females (around 22–25%). In contrast, thefts make up just 12% of male crimes but 39% of female crimes. 

**Table 3.** Summary of number of police records, by offence group and sex.

Offence group	Overall N records (%) (100%=6413)	Males N records (%) (100%=5255)	Females N records (%) (100%=1158)
Arson and criminal damage	807 (12.7)	732 (13.9)	75 (6.5)
Burglary	466 (7.3)	451 (8.6)	15 (1.3)
Drug offences	1237 (19.3)	1095 (20.8)	142 (12.3)
Fraud ^1^	44 (0.7)	-	-
Miscellaneous crimes against society	157 (2.5)	131 (2.5)	26 (2.3)
Possession of weapons	85 (1.3)	77 (1.5)	8 (0.7)
Public order offences	683 (10.7)	572 (10.9)	111 (9.6)
Robbery	102 (1.6)	88 (1.7)	14 (1.2)
Sexual offences ^1^	45 (0.7)	-	n<5
Theft	1077 (16.8)	622 (11.8)	455 (39.3)
Vehicle offences	277 (4.3)	268 (5.1)	9 (0.8)
Violence against the person	1433 (22.4)	1141 (21.7)	292 (25.2)

^1^ Cell counts suppressed to prevent calculation of the small cell count for sexual offence records for females.

## Points to note

### General points on regional police records

Police forces only hold records of crimes committed in their area. Therefore, a lack of an A&SP record does not mean an individual does not have a police record elsewhere. Further, not all crimes are reported to, or recorded by, the police. An additional consideration is that police forces are only able to retain records if there is a justification for doing so. As per MoPI rules, many older records for Category 3 offences where the individual was not involved in any further crime will likely have been deleted and will therefore not have been included in this linkage. Additionally, A&SP used paper records pre-2007 and the majority of these were not transferred to an electronic form. We cannot quantify the extent of deleted records, but we do know that in the pilot linkage of ALSPAC to the PNC there were several pre-2007 records (predominantly in 2005 and 2006) (see Figure 2 in reference 4), meaning we can be sure that some ALSPAC participants did have police records pre-2007. Overall, this means linkage to A&SP records as a way of measuring offending in the ALSPAC cohort will underestimate the total amount of crime committed by this group.

Regional police records do not routinely include data on convictions, and it is important that this is made clear when describing the police data and interpreting findings. While conviction rates can be very high, they do vary by offence type ^13^. The age of criminal responsibility in England is ten years; children below this age cannot be arrested, charged or cautioned if they break the law ^16^. The UK has no statute of limitations for indictable (either-way) and indictable only offences; for summary offences it is generally six months although there are exceptions. (The term ‘statute of limitations’ refers to the maximum time limit after an event that legal proceedings can be initiated: after the time limit has passed, a person cannot be prosecuted regardless of the evidence against them). It is common to see offences, particularly sexual offences, prosecuted many years after the offence took place. 

It is important for researchers using police data to be aware that there are several sources of bias. These include bias in terms of whose criminal behaviour is detected by the police, and the disposal type they are given. Examples of this include the disproportionate use of Stop and Search on Black, Asian and Minority Ethnic communities ^17^ and variations in the rate of reporting of crime across communities and demographic groups ^18^. Bias may also be introduced through the data linkage process if participants with a criminal record are, in general, less active in ALSPAC, resulting in their identifier information ( *e.g.* current name and address) held by the study being out of date; this is likely to be true since levels of participation in ALSPAC are lower among individuals from more deprived backgrounds ^10^ and deprivation is associated with increased involvement in crime ^8, 9^. It is also known that linkage error can be differential with respect to particular socio-demographic characteristics ( *e.g.* non-traditional UK names may be at increased risk of being incorrectly entered into official records) and, finally, missed matches can occur when linking to crime records in particular due to the use of ‘fake’ identifiers. Notwithstanding these limitations, police data have been—and continue to be—a useful, population-level indicator of criminal behaviour.

### Defining an appropriate denominator

As the A&SP records only cover crimes committed in A&S, it is important for researchers to be able to identify who was living in this area so that an appropriate denominator can be defined. Flags have been derived that denote whether an individual was living in A&S on each of their birthdays (this is based on the contact address ALSPAC held for that child’s family at each time point and is unlikely to be completely accurate). At age 10 (the youngest age someone can have a police record in England), almost 90% of the ALSPAC sample for whom there is permission to link to crime data had an address in A&S. This proportion declined only slightly through adolescence but then dropped to 76% by age 24 and 66% by age 28. Overall, over 60% of the sample had an ALSPAC recorded contact address in A&S for every birthday from age 10 through to 28 years. 

### ALSPAC data availability for those with a police record

Participants with an A&SP record have lower response rates to questionnaires and lower attendance rates at clinics than those with no A&SP record ( *i.e.* they have more missing ALSPAC-collected data). This is true at all ages and for most questionnaire types [including mother, partner, child-based (completed by the mother about the child), and child-completed]. Of those participants eligible for crime data linkage (n=12,662), for the vast majority ALSPAC also has permission to link to their health and education records. However, those with a crime record are much less likely to have actively consented to data linkage (given active opt-in consent was only collected where practicable, and this is tied to active study participation) and are much more likely to be non-responders. This emphasises the importance of using opt-out linkage permission approaches and including non-responders in any analyses using linkage data where possible ^19^. 

## Data Availability

If you require further information about the A&SP data, please contact the ALSPAC Data Linkage Team ( alspac-linkage@bristol.ac.uk). ALSPAC data access is through a system of managed open access. The steps below highlight how to apply for access to the data included in this data note and all other ALSPAC data: i. Please read the ALSPAC access policy which describes the process of accessing the data and samples in detail, and outlines the costs associated with doing so. ii. You may also find it useful to browse our fully searchable research proposals database, which lists all research projects that have been approved since April 2011. iii. Please submit your research proposal for consideration by the ALSPAC Executive Committee. You will receive a response within 10 working days to advise you whether your proposal has been approved.
