# Supplementary material for: Linkage of the Avon Longitudinal Study of Parents and Children (ALSPAC) to Avon & Somerset Police regional police records
Source: Wellcome Open Res. 2023 Feb 1;8:47. [Version 1] doi: 10.12688/wellcomeopenres.18720.1 (PMC10403744; doi:10.12688/wellcomeopenres.18720.1)
Supplement: Peer review history

# Peer review: Reviewer response for version 1

**DOI:** 10.21956/wellcomeopenres.20758.r59614

**Published:** 2023-08-04

**Authors:** Shawn Bushway, Nicholas Goldrosen

**Affiliations:** 1 Institute of Criminology, University of Cambridge, Cambridge, England, UK; 2 University at Albany, Albany, New York, USA

This paper describes the valuable task of linking police-recorded offending data from Avon and Somerset (A&S) Police to the broader Avon Longitudinal Study of Parents and Children (ALSPAC) dataset. Administrative data linkages are an increasingly important area of research attention in criminology, such as with the Ministry of Justice’s Data First project [1]. The inclusion of official arrest data in a rich longitudinal dataset like ALSPAC will allow for important new explorations of the role of arrest and justice involvement in the life-course [2]. The linkage process is carefully done and well-described, which will help other UK researchers undertaking administrative data linkages with police data

 While the authors clearly acknowledge many of the linkage’s limitations, some of these deserve closer treatment. 86% of offending in the pilot PNC linkage occurred in the A&S police area -- it is unclear from the pilot linkage paper or this paper whether the non-A&S offending systematically differed from that in A&S [3]. The authors could use the PNC pilot linkage to address whether the offences in the A&S area were similar in age of onset, crime type, and crime severity to the offences recorded by other police forces. This information would be important in understanding criminality that crosses geographic boundaries, such as involvement in county lines or other organised crime [4].

 More importantly, the routine deletion of group 3 offences from local police systems undercuts the ability of this data to show how early arrests for minor events could lead directly to future involvement with the police. This concern is over and above the authors’ observation that this purging, “will underestimate the total amount of crime committed by this group.” The linkage was performed in 2021, when the cohort was aged 29-31 years old, meaning that group 3 offences occurring under the age of 23-25 are vastly underrepresented. The average onset of serious delinquency in other studies has been between 11 and 14 years old; a large portion of desistance occurs in the early 20s [5,6]. Hence, not only the frequency but also the prevalence of offending will be underestimated, as some participants’ offences will likely be wholly excluded from the linked police data. One way to quantify this underrepresentation might be using the pilot PNC linkage, as PNC data is not purged over time. What proportion of participants who do have a police record stemming from the A&S police area in the PNC linkage show no records, or substantially fewer records, in the A&S linked data?

 Additionally, given the importance of self-reported data on criminal offending — and comparisons of this data with official records [7] — the inclusion of some descriptive statistics on self-reported versus officially-recorded crime in the ALSPAC cohort would be helpful. For example, how many ALSPAC participants that self-report criminal justice involvement and did not withdraw consent for linkage have no A&S police record? This answer might also help estimate the impact of purged group 3 offences or offences occurring outside A&S on the linked criminal record data.

 Such a comparison would also place this research into the proper context of other papers that compare self-reported and officially-reported arrests in life-course criminology as a field. Existing studies of this type in the United Kingdom, such as the Cambridge Study in Delinquent Development and the Edinburgh Study of Youth Transitions and Crime, are few [8,9]; the Cambridge study is quite old, with a cohort born in the 1960s. Examples in the U.S. are also rare, and are either dated [10] or limited to youth are already deeply involved with the justice system [11]. The inclusion of police-recorded crime data in a longitudinal study of more recent vintage and from a new geographical area is an important development to understanding the onset and persistence of, and desistance from, offending over the life-course [12] in a world where the discovery of large cohort differences in criminal justice involvement [13-18] are raising serious questions about the supposed constancy of the vaunted age-crime curve [19].

 The inclusion of crime harm in the dataset is another valuable element. Harm indices have become a widely-used tool in both criminological research and practice [20]. The exact origins of the A&S harm score, though, is unclear. Some other crime harm indices have relied on public sentiment [21] or on sentencing guidelines [22], but it is not clear if the A&S scores reflect either of these. It would be helpful to include information on how the A&S harm scores were calculated by police.

 In sum, this paper is a clear and well-written description of a thorough and valuable data linkage project. For criminologists, the inclusion of police data in the ALSPAC dataset makes it an excellent resource — we look forward to additional papers using this important new resource to explore the linkages between arrest and other life events [23] as well as descriptive questions about how the prevalence of police involvement varies between birth cohorts.

 [1] Ministry of Justice (2020), Data First. https://www.gov.uk/guidance/ministry-of-justice-data-first

 [2] Kirk, D. and Wakefield, S. (2018). Collateral Consequences of Punishment: A Critical Review and Path Forward. *Annual Review of Criminology* 1(1), 171-194.

 [3] Boyd, A., Teyhan, A., Cornish, R.P., *et al*. The potential for linking cohort participants to official criminal records: a pilot study using the Avon Longitudinal Study of Parents and Children (ALSPAC) [version 2; peer review: 2 approved]. *Wellcome Open Research *5(271).

 [4] E.g., McLean, R., Robinson, G., and Densley, J.A. (2020). *County lines: criminal networks and evolving drug markets in Britain*. Springer.

 [5] Farrington, D.P. (1986). Age and crime. In Tonry, M. and Morris, N. (eds.) *Crime and Justice*, vol 7. Chicago: University of 189–250.

 [6] Moffitt, T.E. (1993). Adolescence-limited and life-course-persistent antisocial behavior: a developmental taxonomy. *Psychological Review *100(4), 674–701.

 [7] Thornberry, T.P. and Krohn, M.D. (2003). Comparison of Self-Report and Official Data for Measuring Crime. *Measurement Problems in Criminal Justice Research: Workshop Summary*. Washington, DC: The National Academies Press.

 [8] Farrington, D.P., Jolliffe, D., and Coid, J.W. (2021) Cohort profile: the Cambridge study in delinquent development (CSDD). *Journal of Developmental and Life Course Criminology* 7, 278–291

 [9] Smith, D.J., McVie, S., Woodward, R., Shute, J., and McAra, L. (2001). The Edinburgh Study of Youth Transitions and Crime: Key findings at ages 12 and 13. The Edinburgh Study of Youth Transitions and Crime, *Research Digest no. 1.*

 [10] Krohn M. D., Lizotte A. J., Phillips M., Thornberry T. P., Bell K. A. (2013). Explaining systematic bias in self-reported measures: Factors that affect the under- and over-reporting of self-reported arrests. *Justice Quarterly*, 30, 501-528.

 [11] Piquero, A. R., Schubert, C. A., and Brame, R. (2014). Comparing Official and Self-report Records of Offending across Gender and Race/Ethnicity in a Longitudinal Study of Serious Youthful Offenders. *Journal of Research in Crime and Delinquency* 51(4), 526–556.

 [12] E.g., Stewart, A., Dennison, S., Allard, T., *et al*. (2015). Administrative data linkage as a tool for developmental and life-course criminology: The Queensland Linkage Project. *Australian & New Zealand Journal of Criminology* 48(3), 409–428.

 [13] Sampson, R.J. and Smith, L.A. (2021). Rethinking Criminal Propensity and Character: Cohort Inequalities and the Power of Social Change. *Crime and Justice* 50, 13-76.

 [14] Neil, R. and Sampson, R.J. (2021). The Birth Lottery of History: Arrest over the Life Course of Multiple Cohorts Coming of Age, 1995-2018. *American Journal of Sociology* 126(5), 1127–1178.

 [15] Kim, J., Bushway, S., and Tsao, H. (2016). Identifying Classes of Explanations for the Crime Drop: Period and Cohort Explanations in New York State. *Journal of Quantitative Criminology* 32, 357-375.

 [16] Shen, Y., Bushway, S., Sorensen, L., and Smith, H. (2020). Locking Up My Generation: Cohort Differences in Prison Spells Over the Life Course. *Criminology* 54(4), 645-677.

 [17] Bjerk, D. and Bushway, S. (2022). The Long-term Incarceration Consequences of Coming-of-Age in a Crime Boom. *Journal of Quantitative Criminology*.

 [18] Spelman, W. (2022). How cohorts changed crime rates, 1980-2016. Journal of Quantitative Criminology 38(3), 637-671.

 [19] Britt, C.L. (1992) Constancy and Change in the U.S. Age Distribution of Crime: A Test of the ‘Invariance Hypothesis.’ *Journal of Quantitative Criminology* 8(2), 175-87.

 [20] van Ruitenburg, T. and Ruiter, S. (2023). The adoption of a crime harm index: A scoping literature review. *Police Practice and Research* 24(4), 423-445.

 [21] Wolfgang, M., Figlio, R. M., Tracy, P. E., and Singer, S. I. (1985). *The National Survey of Crime Severity*. U.S. Dept. of Justice, Bureau of Justice Statistics.

 [22] Sherman, L., Neyroud, P., and Neyroud, E. (2016). The Cambridge crime harm index: measuring total harm from crime based on sentencing guidelines. *Policing: A Journal of Policy and Practice* 10(3), 171–183.

 [23] Doherty E.E., Cwick, J.M., Green, K.M., and Ensminger, M.E. (2016). Examining the Consequences of the ‘Prevalent Life Events’ of Arrest and Incarceration among an Urban African-American Cohort. Justice Quarterly 33(6), 970-999.

Are sufficient details of methods and materials provided to allow replication by others?

Yes

Is the rationale for creating the dataset(s) clearly described?

Partly

Are the datasets clearly presented in a useable and accessible format?

Yes

Are the protocols appropriate and is the work technically sound?

Yes

Reviewer Expertise:

Criminology

We confirm that we have read this submission and believe that we have an appropriate level of expertise to confirm that it is of an acceptable scientific standard.

## References

[1] : Ministry of Justice: Data First. *UK Government Ministry of Justice/* .2022; Reference source

[2] : Collateral Consequences of Punishment: A Critical Review and Path Forward. *Annual Review of Criminology* .2018;1(1) : 10.1146/annurev-criminol-032317-092045 171-194 10.1146/annurev-criminol-032317-092045

[3] : The potential for linking cohort participants to official criminal records: a pilot study using the Avon Longitudinal Study of Parents and Children (ALSPAC). *Wellcome Open Res* .2020;5: 10.12688/wellcomeopenres.16328.2 271 10.12688/wellcomeopenres.16328.2 36277330PMC9561538

[4] : County Lines. 10.1007/978-3-030-33362-1 10.1007/978-3-030-33362-1

[5] : Age and Crime. *Crime and Justice* .1986;7: 10.1086/449114 189-250 10.1086/449114

[6] : Adolescence-limited and life-course-persistent antisocial behavior: A developmental taxonomy. *Psychological Review* .1993;100(4) : 10.1037/0033-295X.100.4.674 674-701 10.1037/0033-295X.100.4.674 8255953

[7] : Comparison of Self-Report and Official Data for Measuring Crime In Measurement Problems in Criminal Justice Research: Workshop Summary. *Washington, DC: The National Academies Press, National Academies of Sciences, Engineering, and Medicine* .2003; 10.17226/10581 43-94 10.17226/10581 Reference source

[8] : Cohort Profile: The Cambridge Study in Delinquent Development (CSDD). *Journal of Developmental and Life-Course Criminology* .2021;7(2) : 10.1007/s40865-021-00162-y 278-291 10.1007/s40865-021-00162-y

[9] : The Edinburgh Study of Youth Transitions and Crime: Key Findings at Ages 12 and 13. *Centre for Law and Society, University of Edinburgh* .2001; Reference source

[10] : Explaining Systematic Bias in Self-Reported Measures: Factors that Affect the Under- and Over-Reporting of Self-Reported Arrests. *Justice Quarterly* .2013;30(3) : 10.1080/07418825.2011.606226 501-528 10.1080/07418825.2011.606226

[11] : Comparing Official and Self-report Records of Offending across Gender and Race/Ethnicity in a Longitudinal Study of Serious Youthful Offenders. *Journal of Research in Crime and Delinquency* .2014;51(4) : 10.1177/0022427813520445 526-556 10.1177/0022427813520445

[12] : Administrative data linkage as a tool for developmental and life-course criminology: The Queensland Linkage Project. *Australian & New Zealand Journal of Criminology* .2015;48(3) : 10.1177/0004865815589830 409-428 10.1177/0004865815589830

[13] : Rethinking Criminal Propensity and Character: Cohort Inequalities and the Power of Social Change. *Crime and Justice* .2021;50(1) : 10.1086/716005 13-76 10.1086/716005

[14] : The Birth Lottery of History: Arrest over the Life Course of Multiple Cohorts Coming of Age, 1995–2018. *American Journal of Sociology* .2021;126(5) : 10.1086/714062 1127-1178 10.1086/714062

[15] : Identifying Classes of Explanations for Crime Drop: Period and Cohort Effects for New York State. *Journal of Quantitative Criminology* .2016;32(3) : 10.1007/s10940-015-9274-5 357-375 10.1007/s10940-015-9274-5

[16] : Locking up my generation: Cohort differences in prison spells over the life course. *Criminology* .2020;58(4) : 10.1111/1745-9125.12256 645-677 10.1111/1745-9125.12256

[17] : The Long-Term Incarceration Consequences of Coming-of-Age in a Crime Boom. *Journal of Quantitative Criminology* .2022; 10.1007/s10940-022-09559-4 10.1007/s10940-022-09559-4

[18] : How Cohorts Changed Crime Rates, 1980–2016. *Journal of Quantitative Criminology* .2022;38(3) : 10.1007/s10940-021-09508-7 637-671 10.1007/s10940-021-09508-7

[19] : Constancy and change in the U.S. age distribution of crime: A test of the “invariance hypothesis”. *Journal of Quantitative Criminology* .1992;8(2) : 10.1007/BF01066743 175-187 10.1007/BF01066743

[20] : The adoption of a crime harm index: A scoping literature review. *Police Practice and Research* .2023;24(4) : 10.1080/15614263.2022.2125873 423-445 10.1080/15614263.2022.2125873

[21] : National Survey of Crime Severity. *Bureau of Justice Statistics (BJS), University of Pennsylvania* .1985; Reference source

[22] : The Cambridge Crime Harm Index: Measuring Total Harm from Crime Based on Sentencing Guidelines. *Policing* .2016;10(3) : 10.1093/police/paw003 171-183 10.1093/police/paw003

[23] : Examining the Consequences of the "Prevalent Life Events" of Arrest and Incarceration among an Urban African-American Cohort. *Justice Q* .2016;33(6) : 10.1080/07418825.2015.1016089 970-999 10.1080/07418825.2015.1016089 27616814PMC5015121

# Peer review: Reviewer response for version 1

**DOI:** 10.21956/wellcomeopenres.20758.r59630

**Published:** 2023-06-29

**Authors:** Stacy Tzoumakis

**Affiliations:** 1 School of Criminology and Criminal Justice, Griffith University, Nathan, Queensland, Australia; 2 Discipline of Psychiatry and Mental Health, University of New South Wales, Sydney, New South Wales, Australia

In this Data Note, the authors provide detailed information on the linkage of regional police records to the participants of the Avon Longitudinal Study of Parents and Children. This will be an invaluable resource to answer questions about early life risk and protective factors linked to criminal justice involvement in adolescence and adulthood. They provide clear and concise information on the procedures and record linkage methods. The deterministic and probabilistic record linkage methods used were appropriate. The authors also acknowledge the limitations and difficulties with conducting record linkage with police data such as the use of aliases.

 Many record linkage studies can provide a rate of false-positive linkages (e.g., n/1000 persons or %) in the data to better demonstrate the accuracy of the linkage. If it is possible to do so, the authors should consider adding this information. Moreover, the authors should consider adding the number of records and prevalence for the domestic abuse indicator (perhaps to Table 3?) as this will surely be of interest to potential collaborators.

 A few other minor comments:

1. In the Abstract, the authors state: “ *In total, ALSPAC had permission to link to crime records for 12,662 of the ‘study children’ (now adults, who were born in the early 1990s).*” They should consider providing the denominator of the study children (n~14,000) or the % to demonstrate that this was relatively high.
2. In the first sentence of the background, the authors refer to upstream risk factors. It might be good to provide some examples or define this for the readership.
3. On p. 3 the authors state that “ *pursuing linkage to local police records would be a more targeted yet equally valid approach*”. I would consider rewording “equally valid” as they acknowledge later that using this data source does not account for convictions. It has other strengths such as being a broader measure and an indicator of earlier contact with the CJS.
4. On p. 5, it would be good to provide the total number of twins in the ALSPAC data for context.
5. On p. 6, consider providing the n to specify what consists of ‘a few’ in this sentence: “ *A few 'true' links also had some of the lowest confidence scores…*”
6. On p. 6, in Stage 2: Extracting attribute data, the authors should provide percentages or ranges to describe what is meant by the ‘majority’ and ‘high’ in these two sentences: “ *majority will go on to face trial in court*”; “ *Conviction rates are high for many offences…*”
7. It was great to see the brief summary of the data include numbers by sex, which already revealed important differences. I was surprised to see that violence against the person had the highest prevalence, especially for females. Violence is usually the least common crime type. Traffic and other less serious offences are typically highest in population studies. It would be helpful to have a more detailed description of what specific offence types (common assault? acts intended to cause injury?) were included in this offence group to unpack this finding.
8. On page 8, consider providing some numbers to show what is meant by ‘very high’: “ *While conviction rates can be very high…*”

Are sufficient details of methods and materials provided to allow replication by others?

Yes

Is the rationale for creating the dataset(s) clearly described?

Yes

Are the datasets clearly presented in a useable and accessible format?

Yes

Are the protocols appropriate and is the work technically sound?

Yes

Reviewer Expertise:

Criminology, child maltreatment, child development, administrative record linkage

I confirm that I have read this submission and believe that I have an appropriate level of expertise to confirm that it is of an acceptable scientific standard.

# Peer review: Reviewer response for version 1

**DOI:** 10.21956/wellcomeopenres.20758.r58111

**Published:** 2023-06-27

**Authors:** Stephanie D'Souza

**Affiliations:** 1 The University of Auckland, Auckland, Auckland, New Zealand

The data note reviewed details the linkage of ALSPAC and regional police records. There is increasing use of administrative records, beyond just the public health space, and into areas of social sciences. This development has made significant contributions to our understanding of key social outcomes. Linkage to prospective surveys has been identified as a significant avenue for future research (e.g. see Milne et al. 2022, *Annual Review of Developmental Psychology*); therefore, the resource described in this data note can certainly provide significant value.

 In general, the methodological processes appear appropriate and have been outlined clearly. Importantly, clear measures have been taken to prevent the possibility of identification due to linkage. Caveats with the data have also been addressed.

 I do have two questions about some of the methodological processes that could be clarified in the text where appropriate:

1. Does the linkage method allow for phonetic similarity between names? (e.g. Li and Lee)
2. Regarding the manual review of links - does this mean links that met the three criteria specified on page 5 were retained? I’m concerned about the full postcode match being the only criteria for retention in that case, especially given that individuals may have moved and not updated information - particularly those who are likely to have a police record. What % of links met this criterion?

Finally, regarding the disposal outcome types, the authors note that conviction data aren’t available and can differ based on offence type. Are there published statistics on the average conviction rate or range in conviction rate by offence type that could be specified here?

Are sufficient details of methods and materials provided to allow replication by others?

Partly

Is the rationale for creating the dataset(s) clearly described?

Yes

Are the datasets clearly presented in a useable and accessible format?

Yes

Are the protocols appropriate and is the work technically sound?

Yes

Reviewer Expertise:

Longitudinal research; administrative data; maternal mental health; child development

I confirm that I have read this submission and believe that I have an appropriate level of expertise to confirm that it is of an acceptable scientific standard, however I have significant reservations, as outlined above.

## References

[1] : Use of Population-Level Administrative Data in Developmental Science. *Annu Rev Dev Psychol* .2022;4(1) : 10.1146/annurev-devpsych-120920-023709 447-468 10.1146/annurev-devpsych-120920-023709 37284522PMC10241456
